# The hepatic pre-metastatic niche in pancreatic ductal adenocarcinoma

**DOI:** 10.1186/s12943-018-0842-9

**Published:** 2018-06-14

**Authors:** Demi S. Houg, Maarten F. Bijlsma

**Affiliations:** 1 Laboratory for Experimental Oncology and Radiobiology, Center of Experimental and Molecular Medicine, Cancer Center Amsterdam and Academic Medical Center, Amsterdam, the Netherlands; 2 Oncode Institute, Academic Medical Center, Amsterdam, the Netherlands

**Keywords:** Pancreatic cancer, Metastasis, Niche, Stroma

## Abstract

Pancreatic ductal adenocarcinoma (PDAC) remains one of the most aggressive malignancies to date, largely because it is associated with high metastatic risk. Pancreatic tumors have a characteristic tendency to metastasize preferentially to the liver. Over the past two decades, it has become evident that the otherwise hostile milieu of the liver is selectively preconditioned at an early stage to render it more conducive to the engraftment and growth of disseminated cancer cells, a concept defined as pre-metastatic niche (PMN) formation. Pancreatic cancer cells exploit components of the tumor microenvironment to facilitate their migration out of the primary tumor, which often involves conversion of pancreatic cancer cells from an epithelial to a mesenchymal phenotype via the epithelial-to-mesenchymal transition. Pancreatic stellate cells and matrix stiffness have been put forward as major drivers of invasiveness in PDAC. Even before the onset of pancreatic cancer cell dissemination, soluble factors and extracellular vesicles secreted by the primary tumor, and possibly even premalignant lesions, help shape a supportive niche in the liver by providing vascular docking sites for circulating tumor cells, enhancing vascular permeability, remodeling the extracellular matrix and recruiting immunosuppressive inflammatory cells. Emerging evidence suggests that some of these tumor-derived factors may represent powerful diagnostic or prognostic biomarkers. Though our understanding of the mechanisms driving PMN formation in PDAC has expanded considerably, many outstanding questions and challenges remain. Further studies dissecting the molecular and cellular events involved in hepatic PMN formation in PDAC will likely improve diagnosis and open new avenues from a therapeutic standpoint.

## Background

Metastatic spread of malignant cells continues to pose a problem to the treatment of cancer and remains the principal cause of cancer mortality, particularly in the case of pancreatic ductal adenocarcinoma (PDAC). The majority of patients with PDAC presents with systemic disease at the time of diagnosis and only 8% of patients survive for 5 years or longer following diagnosis [1]. Although surgical resection of the primary tumor remains the most effective strategy to extend patient survival, 85–90% of patients are considered ineligible owing to the systemic nature of the disease and a failure to detect the disease early [1–3]. Moreover, with more than 80% of patients suffering systemic spread following surgery, disease relapse is often the rule rather than the exception [4]. Our increased understanding of pancreatic cancer biology [5–10] and advances in therapeutic approaches [11–13] have yet to translate into significant improvements in overall survival. Until then, the development of new treatments directed at metastatic spread of pancreatic cancer cells may prove particularly valuable, underscoring the need for further research into the pathogenesis of pancreatic cancer metastasis.

A growing body of research has demonstrated that cancer cells metastasize to the milieu of specific organs with a predetermined selectivity. The concept of organ selectivity of metastasis, or organotropism, was first introduced by Stephen Paget in 1889 in the form of the seed and soil hypothesis [14]. Based on the non-random distribution of metastases in patients with breast cancer, he hypothesized that cancer cells (the “seeds”) home exclusively to organs that are conducive to their survival and growth (the fertile “soil”). Autopsy studies investigating the localization of metastases in patients with PDAC have revealed that pancreatic carcinoma cells metastasize disproportionately to the liver. Liver metastases are found in 76–80% of patients; other common sites of metastasis include the peritoneum (48%) and the lungs (45%) [15, 16].

Many efforts in the field of metastasis have centered on molecular and genetic alterations that determine the metastatic capacity of certain tumor cells. However, as the efficiency of metastasis is limited by continuous immune surveillance, apoptosis and other barriers [17], tumor cell-intrinsic properties alone are insufficient for successful metastatic seeding and outgrowth [18]. Our understanding of metastasis has advanced significantly in recent years and various fundamental discoveries have added an additional layer of complexity to the original seed and soil hypothesis. There is a growing appreciation that primary tumors precondition the microenvironments of future sites of metastasis to form so-called pre-metastatic niches (PMNs) that support the engraftment, survival and outgrowth of circulating tumor cells even prior to their arrival [18–21]. The development of PMNs is governed by a complex series of reciprocal interactions between tumor cells and various components of the tumor microenvironment, as well as the exploitation of resident and recruited cells in secondary target organs. The current review aims to describe the emerging knowledge base with regard to mechanisms involved in the formation of the hepatic PMN and subsequent metastasis in PDAC, and highlights some of the opportunities and challenges regarding the translation of these mechanistic insights into promising diagnostic and therapeutic approaches. To this end, we will first discuss several cues from the tumor microenvironment that prompt pancreatic cancer cells to migrate out of the primary tumor, before moving on to the extrinsic and intrinsic factors that help shape a permissive niche in the liver for colonization by disseminated tumor cells.

### The role of the epithelial-to-mesenchymal transition in the metastatic cascade

Metastasis is a multistep process involving localized migration, intravasation into and transport through the lymphatic system or the systemic circulation, followed by extravasation at distant capillaries, the development of micrometastases, and ultimately macrometastatic colonization [22]. Hence, a crucial step in the metastatic cascade is the acquisition of migratory and invasive properties. This requires pancreatic cancer cells to exchange many of their epithelial characteristics, such as apical-basal polarity and cell-cell adhesion, for mesenchymal traits via a cellular program known as epithelial-to-mesenchymal transition (EMT) [23]. Though first identified in the context of embryonic morphogenesis [24], in recent years EMT has also become prominently implicated in wound resolution, fibrosis, tissue regeneration and cancer progression [25]. Molecular changes associated with this transition include the loss of epithelial markers like E-cadherin, cytokeratins, occludin and claudin, and the gain of mesenchymal markers such as N-cadherin, vimentin and fibronectin [25, 26]. In addition, cells that have switched to the mesenchymal state adopt a spindle-like shape rather than the columnar shape that is typical of epithelial cells. The mesenchymal phenotype following induction of EMT is characterized by enhanced migratory capacity, invasiveness, increased resistance to apoptosis and elevated production of extracellular matrix (ECM) components [25].

The functional role of EMT in invasion and metastasis has recently been challenged by Zheng and colleagues, who reported that rates of metastases in genetically engineered mouse models of PDAC were not affected by knockout of *Snai1* or *Twist1*, two genes encoding EMT-inducing transcription factors [27]. Consequently, the authors drew the contentious conclusion that EMT is dispensable for metastatic dissemination of pancreatic cancer cells. Recently, however, Aiello and co-workers highlighted several caveats that cast doubt on the validity of their claims, including the fact that the study involved the use of a marker of EMT deemed unreliable in the selected mouse model [28]. Secondly, EMT is a highly complex cellular program that is regulated through multiple molecular pathways, some of which remain incompletely understood. Genetic deletion of either *Snai1* or *Twist1* may have failed to effectively abolish all manifestations of EMT. This is corroborated by the finding that conditional knockout of another EMT activator, Zeb1, in the same mouse model in fact strongly influences malignant progression of PDAC [29]. Knockout of *Zeb1* had detrimental effects on cell plasticity and fixed pancreatic tumor cells in an epithelial state. This was accompanied by a remarkable reduction in local invasion in the primary tumor as well as in the capacity of tumor cells to colonize and metastasize to distant organs, which is in sharp contrast to the findings of Zheng et al. following *Snai1* or *Twist1* depletion. Krebs and colleagues concluded that different EMT transcription factors may have distinct and tissue-specific functions that are complementary rather than redundant. In this regard, Snai1 and Twist1 may indeed be dispensable for metastatic progression of PDAC, but the critical role of the EMT activator Zeb1 in this cancer type means that we cannot dismiss EMT as a fundamental event preceding invasion and metastasis of pancreatic cancer [25].

Nonetheless, the study by Zheng et al. did uncover an unexpected relationship between *Snai1*- and *Twist1*-dependent EMT and chemotherapy resistance. It was already demonstrated in a previous study that Zeb1-mediated EMT contributes to resistance to gemcitabine, 5-fluorouracil and cisplatin in various pancreatic cancer cell lines [30]. Knockdown of Zeb1 increased the expression of epithelial markers and restored drug sensitivity. Zheng et al. elaborated on these findings by showing that knockout of *Snai1* or *Twist1* in a KPC mouse model of PDAC correlated with an increase in drug sensitivity and overall survival of gemcitabine-treated mice [27]. Enhanced drug sensitivity coincided with upregulated expression of drug transporters, providing a potential mechanistic underpinning for the link between EMT and chemoresistance in PDAC. These data further accentuate the eminent role of EMT in the progression of PDAC.

### Support from the stroma in tumor cell migration and invasion

The acquisition of migratory and invasive properties is not only driven by genetic perturbations that have evolved during tumor development. Rather, the interplay between tumor cells and their microenvironment represents another critically important driver of tumor cell invasion and metastasis. Histologically, a prominent characteristic of PDAC is the extensive fibrotic response surrounding neoplastic cells (also known as desmoplasia or tumor-associated stroma), which may constitute up to 80% of the total tumor volume [31, 32]. In the stroma of normal epithelial tissues, tissue homeostasis is maintained by a dynamic network of fibroblasts, inflammatory cells, ECM and vasculature composed of endothelial cells and pericytes [33]. By contrast, in the stroma surrounding pancreatic cancer tissue, these cellular and acellular components are conscripted and corrupted by pancreatic cancer cells to form a tumor-promoting environment which stimulates cancer cell proliferation [34, 35] and migration [36, 37], and serves as a reservoir for cytokines and growth factors [38]. Furthermore, the tumor-associated stroma in PDAC forms a barrier to the delivery of multiple therapeutic agents [39, 40] and conveys chemo- and radioresistance [34, 41]. In the following section, we will discuss how the stroma in PDAC spurs pancreatic cancer cells to migrate out of the primary tumor to set the stage for systemic spread.

#### Dynamic interplay between activated pancreatic stellate cells and pancreatic cancer cells drives malignant behaviour

The principal cells responsible for PDAC-associated fibrogenesis are activated fibroblasts or myofibroblasts, the most abundant cell type in the desmoplastic stroma of PDAC [42]. While these myofibroblasts may also originate from pancreas-resident fibroblasts or from bone marrow-derived mesenchymal stem cells, a prominent source of myofibroblasts in PDAC are pancreatic stellate cells (PSCs). Repeated or sustained pancreatic injury causes the transformation of normally quiescent PSCs into aberrantly activated, fibroblastic cells, leading to enhanced deposition of ECM proteins such as collagen and fibronectin [33]. Clinically, there is an inverse correlation between the extent of PSC activation and prognosis in PDAC patients, highlighting the role of PSCs in pancreatic cancer progression [32].

Both in vitro and in vivo studies have revealed the existence of an intricate bidirectional crosstalk between pancreatic cancer cells and PSCs which fosters both local tumor growth and distant metastasis. Pancreatic cancer cells promote the chemotactic recruitment of PSCs to their vicinity via the secretion of platelet-derived growth factor (PDGF) [43, 44]. Simultaneously, PDGF has direct mitogenic effects on PSCs and thus stimulates their proliferation [45]. Pancreatic cancer cells also release fibrogenic mediators such as transforming growth factor β1 (TGFβ1) which stimulate the synthesis and deposition of ECM components by activated PSCs. In turn, PSCs contribute to pancreatic cancer cell survival by promoting their proliferation, suppressing apoptosis and possibly protecting them from chemo- and radiotherapy [34, 44]. In a recent study, cancer-associated fibroblasts (CAFs) and PSCs were found to constitute not merely a biophysical barrier to gemcitabine delivery but in fact take up the drug and trap its active metabolite intracellularly as a result of downregulated expression of gemcitabine-inactivating enzymes and transporters, thereby reducing the amount of drug available to cancer cells [46].

In the context of metastasis, PSCs have emerged as one of the key effector cells in the shift of pancreatic cancer cells toward the more motile, mesenchymal state. Pancreatic cancer cells co-cultured with human PSCs (hPSCs) assume a fibroblastic morphology, and exhibit transcriptional downregulation of the epithelial markers E-cadherin and cytokeratin 19 and upregulation of the mesenchymal markers vimentin and Snai1, concomitant with a marked increase in pancreatic cancer cell motility [47]. In an attempt to recapitulate the influence of the stromal compartment on tumor progression in vivo, Hwang et al. used an orthotopic mouse model of PDAC to investigate the incidence of metastasis. Co-injection of human pancreatic cancer cells and hPSCs into mice resulted in an increase in the number of distant metastases proportional to the relative number of stellate cells present in the mixture. Distant metastases could be found even when a limiting number of cancer cells was injected, further emphasizing that the dynamic interplay between pancreatic cancer cells and PSCs creates a favorable microenvironment that nourishes malignant behavior.

There have been multiple endeavors to delineate the molecular mechanisms underlying the influence of PSCs on the invasive capacity of pancreatic cancer cells. Given that conditioned medium from isolated hPSCs significantly enhances pancreatic cancer cell invasion, the observed pro-metastatic effects of PSCs appear to be mediated largely by soluble factors [34]. One factor that has been intensively studied with respect to pancreatic cancer cell invasion is stromal-derived factor-1 (SDF-1). Secretion of TGFβ by pancreatic cancer cells can stimulate SDF-1 production and secretion by PSCs via the upregulation of galectin-1 [48]. Pancreatic cancer cells express high levels of the SDF-1 receptor C-X-C chemokine receptor 4 (CXCR4) [49]. Interaction of SDF-1 with CXCR4 induces EMT in pancreatic cancer cells, allowing them to migrate along gradients of SDF-1 generated by PSCs. In another study, the presence of stromal fibroblasts increased pancreatic cancer cell expression of a set of genes linked to tumor cell invasion, including *cyclooxygenase 2* (*COX-2*)/*prostaglandin-endoperoxide synthase 2* (*PTGS2*), *hyaluronan synthase 2* (*HAS2*), and *matrix metalloproteinase-1* (*MMP-1*) [50]. Constitutive expression of COX-2 has previously been shown to increase the metastatic potential of human colorectal cancer cells in vitro [51]. The increased invasiveness of these cells was associated with elevated activation of MMP-2, which mediates proteolytic degradation of the ECM and thus facilitates cancer cell migration [52]. A similar role for COX-2 overexpression in PDAC seems plausible, as it has been reported that downregulation of COX-2 expression represses tumor cell invasiveness and motility in various pancreatic cancer cell lines and reduces the number of liver metastases in an orthotopic mouse model of PDAC [53, 54].

Hyaluronan is a glycosaminoglycan that is markedly enriched in the ECM surrounding pancreatic tumors [55], and has previously been reported to stimulate motility and invasion in cell culture models of fibrosarcoma [56], colon cancer [57] and prostate cancer [58]. In PDAC, tumor-associated stromal fibroblasts induce the expression of *HAS2* in pancreatic cancer cells, thereby enhancing the rate of synthesis and deposition of hyaluronan [50]. Subsequent accumulation of hyaluronan in the tumor microenvironment stimulates pancreatic cancer cell motility [37] and enhances the frequency of metastasis [59]. The stimulatory effect of hyaluronan on cell motility may be linked to its centrally important role in EMT. In mice, a lack of hyaluronan during embryonic development abrogates the ability of cardiac endothelial cells to undergo EMT and disrupts cardiac morphogenesis [60]. Furthermore, overproduction of hyaluronan induces EMT in non-transformed epithelial cells, with increased anchorage-independent growth and invasiveness as a result [61]. In pancreatic cancer cells, increased production of hyaluronan leads to loss of E-cadherin and cytoplasmic accumulation of β-catenin, suggesting that hyaluronan may promote pancreatic cancer cell motility by inducing EMT [62].

The formation of new blood vessels is an integral hallmark of cancer [63] and not only provides tumor sustenance in the form of oxygen and nutrients, but could also represent a way to increase the potential for metastatic spread [64]. PSCs are believed to interact closely with endothelial cells in order to promote angiogenesis. By secreting pro-angiogenic mediators such as vascular endothelial growth factor (VEGF), PSCs can induce tube formation in vitro [65, 66] and increase the population of endothelial cells in an orthotopic mouse model of PDAC [66]. It is worth noting, however, that the central areas of pancreatic tumors are notoriously hypovascularized and thus highly hypoxic, while a higher degree of vascularity is evident at the periphery of the tumor [39]. Nonetheless, hypoxia has long been recognized to elicit malignant behavior and actually serves as a cue for VEGF secretion and the induction of pro-angiogenic responses by PSCs [65, 67]. Thus, perhaps tumor hypoxia triggers PSCs to instruct endothelial cells to generate new capillary beds specifically at the invasive front of the tumor [68], with the aim to facilitate proliferation and metastatic dissemination of pancreatic cancer cells.

Interestingly, cells positive for α-smooth muscle actin (αSMA) – a marker of activated PSCs – and even PSCs themselves have been detected in distant metastases [44, 66, 69], which calls into question the common perception that systemic spread is solely restricted to cancer cells. In fact, PSCs have been observed to migrate through an endothelial cell monolayer in response to pancreatic cancer cell-derived PDGF [66]. Another study using an organotypic model of PDAC showed that nuclear translocation of fibroblast growth factor 2 (FGF2) and fibroblast growth factor receptor 1 (FGFR1) in PSCs drives invasion of both PSCs and pancreatic cancer cells into the surrounding ECM [70]. In tissue sections of PDAC, myofibroblasts with nuclear FGF2 and FGFR1 are abundant at the invasive front but not in central areas of the tumors. These data support a model in which PSCs actually have the capacity to co-migrate with pancreatic cancer cells to future metastatic sites, where they could potentially assist their seeding, survival and proliferation.

#### Matrix stiffness as a pro-migratory cue

More recently, biomechanical properties of the stromal compartment have gained increasing interest as mediators of tumor cell behaviour in PDAC. The ECM comprises a diverse array of macromolecules including collagens, fibronectin and proteoglycans, the concentrations and post-translational modifications of which are normally tightly controlled [71]. Disruption of ECM homeostasis during tumor progression – as a result of persistent activation of PSCs – is characterized by increased deposition, altered organization and crosslinking of ECM components, giving rise to a particularly rigid matrix. Though the ECM has historically been regarded as merely a structural scaffold and barrier to tumor cell migration, it is now increasingly acknowledged that matrix stiffness associated with the desmoplastic reaction can provide biomechanical cues that modulate intracellular signaling pathways and augment tumor malignancy.

Sensing of mechanical forces by cells is mediated by integrins. In response to exogenous forces imposed on cells, integrins aggregate into focal adhesion complexes at the cell membrane to initiate downstream signaling events, thereby transducing mechanical inputs into biochemical outputs [72, 73]. In a variety of pancreatic cancer cell lines, extracellular stiffness is associated with elevated expression of vimentin, reduced expression of E-cadherin, and nuclear localization of β-catenin, YAP and TAZ, changes known to be linked to EMT [74]. The extent to which pancreatic cancer cells undergo EMT is dependent on the degree of matrix rigidity. Gradual increases in matrix rigidity parallel with tumor progression promote mesenchymal behavior in a progressive manner, illustrating that EMT is not just a binary switch between two cellular phenotypes, but rather a highly plastic transition. The molecular pathway underlying stiffness-induced EMT has recently been explored in mammary epithelial cells and involves a potential role for Twist1 as a mechanotransducer [75]. In the absence of mechanical stimuli, this EMT-associated transcription factor is retained in the cytoplasm by G3BP2. In response to matrix stiffness and subsequent integrin activation, the interaction between Twist1 and G3BP2 is disrupted through phosphorylation of a tyrosine residue within the G3BP2-binding motif of Twist1, allowing Twist1 to enter the nucleus and induce an EMT transcriptional program. Whether a similar mechanotransduction pathway drives stiffness-induced EMT in PDAC remains a matter of speculation.

Besides adopting pro-migratory mesenchymal properties, pancreatic cancer cells respond to stiff microenvironments by enhancing cellular contractility through the Rho/ROCK pathway downstream of integrin activation [72, 75]. This shift to a contractile state is associated with augmented activity of matrix metalloproteinases (MMPs), a family of endopeptidases which functions predominantly to degrade matrix proteins [75]. A dense and highly crosslinked ECM represents a physical barrier that needs to be compromised in order for tumor cells to migrate out of the primary tumor and intravasate into blood vessels. Thus, it has been postulated that pancreatic cancer cells tactfully tune MMP activity according to the stiffness of their surroundings to facilitate their emigration [75].

On the other hand, dynamic regulation of MMP activity is also required to prevent excessive degradation of the ECM, as type I collagen secreted from PSCs plays a key role in pancreatic cancer cell motility. Though collagen I is a well-established mediator of EMT in pancreatic cancer cells [76–78], it also orchestrates pro-migratory mechanisms that are much less time-consuming. Carcinoma cells exploit thickened, linearized collagen bundles of the ECM as tracks for directional migration within the tumor [71]. Indeed, migration of carcinoma cells in highly metastatic breast tumors appears to be guided by collagen fibres [79]. Likewise, pancreatic cancer cells interact with PSC-secreted collagen I via the α2/β1 integrin-focal adhesion kinase (FAK) signaling pathway and use it directly as a low-resistance scaffold to adhere to and migrate along [80]. Furthermore, alignment of collagen fibres has been observed in PDAC in vivo [81], and intravital microscopy of pancreatic tumors has shown migratory cells orienting their protrusions toward collagen fibres [82], suggesting an important role for stiffened collagen fibres in pancreatic cancer metastasis.

### Preparing the soil: Primary tumor-derived factors drive hepatic PMN formation

**Fig. 1:**
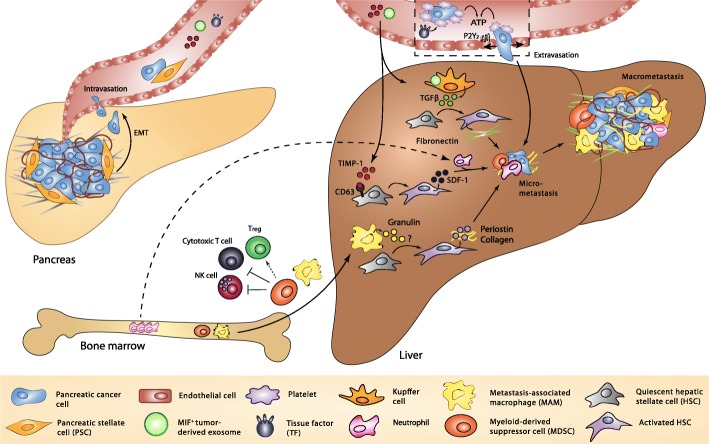
Molecular and cellular events involved in PMN formation in PDAC. PSCs and matrix stiffness, part of the tumor-associated stroma, assist invasion and intravasation of pancreatic cancer cells, for example by inducing EMT. Meanwhile in the hepatic microenvironment, platelet activation and thrombus formation in response to microparticle-associated TF secreted by the primary tumor facilitates the arrest of disseminated pancreatic cancer cells. Opening of the endothelial barrier by activated platelets then facilitates their extravasation. Exosomes enriched in MIF shed by pancreatic cancer cells interact with Kupffer cells, which activate HSCs via the release of TGFβ. The deposition of fibronectin by activated HSCs provides docking sites for metastasis-promoting bone marrow-derived cells such as macrophages and MDSCs. MDSCs impose immune tolerance by inhibiting NK cells and cytotoxic T cells and by promoting the development of Treg. Pancreatic cancer cell-derived TIMP-1 activates HSCs via its cognate receptor CD63 and induces the secretion of SDF-1, a chemoattractant for bone marrow-derived neutrophils. HSCs are also activated in response to MAM-derived granulin, which induces the production of periostin and collagen by HSCs. MAMs also inhibit anti-tumor immune responses by taking on an immunosuppressive M2 phenotype. PSC, pancreatic stellate cell; EMT, epithelial-to-mesenchymal transition; TF, tissue factor; MIF, macrophage inhibitory factor; HSC, hepatic stellate cell; TGFβ, transforming growth factor β; MDSC, myeloid-derived suppressor cell; NK cell, natural killer cell; Treg, regulatory T cell; TIMP-1, tissue inhibitor of metalloproteinases-1; CD63, cluster of differentiation 63; SDF-1, stromal-derived factor-1; MAM, metastasis-associated macrophage

Once pancreatic cancer cells have acquired the capacity to invade, systemic spread is underway. Egress from the primary tumor, however, is only the first hurdle to clear on the way to successful metastasis. Mouse models of PDAC have demonstrated that up to 68% of pancreatic cancer cells are capable of fully completing an EMT program, and, more strikingly, that circulating tumor cells can be detected even before a detectable tumor arises at the primary site [83]. Despite this, no more than 0.01% of cells ultimately end up establishing metastatic outgrowths [84], which goes to show just how inefficient the process of metastasis really is. Therefore, it is of paramount importance that the primary tumor preconditions the otherwise hostile microenvironment of the secondary site such that it can sustain metastatic colonization. The stepwise evolution of the hepatic PMN is initiated early during the progression of PDAC, possibly already during premalignant phases. Given that research into hepatic PMN development in PDAC is still in its infancy, the following section will not encompass an exhaustive list of pro-metastatic mediators. Rather, this section aims to provide a detailed understanding of how tumor-secreted factors in PDAC contribute to the metastatic success of disseminated pancreatic cancer cells by transforming the liver into a hospitable niche (Fig. 1), with a focus on tissue factor (TF), tissue inhibitor of metalloproteinases-1 (TIMP-1) and exosomes.

#### Tissue factor-induced thrombus formation shows circulating pancreatic cancer cells the way out

Pancreatic cancer patients have long been recognized to be at a higher risk of venous thromboembolism [85]. Platelets are key players in thrombosis, and many studies have linked platelet count and activity to metastatic potential and poor outcome in PDAC [86–91]. Tumor cells have been shown to interact closely with platelets and induce their aggregation in favor of metastasis. Pancreatic tumor cells are capable of eliciting a remote pro-thrombotic state via the release of microparticles expressing TF into the systemic circulation [92–96]. TF is a prime initiator of the coagulation cascade; complex formation of TF with coagulation factor VII (FVII) triggers a series of reactions that culminate in the generation of thrombin and fibrinogen, which mediate platelet activation and aggregation into a fibrin clot [97]. The accumulation of platelets and the formation of thrombi could potentially be one of the initial hallmarks of PMN development [18]. Thrombus formation reduces shear stress at the vessel walls and interstitial fluid flow, so by inducing thrombosis in the hepatic vasculature, the primary tumor in the pancreas may remotely guide the arrest and subsequent extravasation of dissociated pancreatic tumor cells at the pre-metastatic site. Moreover, TF-induced platelet aggregates are also a major source of chemotactic signals and promote the homing of myeloid cells, for example granulocytes and monocytes/macrophages, to early metastatic niches [98, 99]. Myeloid cells are vital components of a developing PMN and will be discussed later in this review (see “Systemic immune cell recruitment”). TF-expressing microparticles shed from pancreatic tumor cells have also been shown to directly convert quiescent endothelial cells to a pro-adhesive and pro-inflammatory phenotype by upregulating the adhesion molecule E-selectin and inducing secretion of interleukin-8 (IL-8) [100]. Shortly after vascular arrest of pancreatic tumor cells, TF-activated platelets may further expedite their migration across the endothelium of hepatic blood vessels by secreting adenine nucleotides, which engage P2Y_2_ receptors on endothelial cells and instigate the opening of the endothelial barrier [101].

In addition, fibrinogen deposition and platelet activation induced by tumor-derived TF prompts the formation of platelet-tumor cell aggregates [102–104]. Besides further enhancing the arrest rate of tumor cells at distal vessels through multiple adhesion molecules, such aggregates may also physically shield pancreatic tumor cells from hematogenous shear forces and destruction by cytolytic natural killer (NK) cells.

#### Tissue inhibitor of metalloproteinases-1: A double-edged sword in metastasis

Given that proteolytic remodeling of the ECM is an indispensable aspect of systemic spread of tumor cells, MMPs have been widely regarded to serve pro-invasive and pro-metastatic functions [105]. However, the fact that a growing number of MMPs were found to have tumor-suppressive properties soon dampened enthusiasm for this notion. Perhaps even more paradoxical seemed to be the finding that an increase in systemic levels of TIMP-1, an endogenous inhibitor of a range of MMP family members, in fact renders the liver more susceptible to metastasis [106]. These and other observations indicated that the biology of MMPs and their respective inhibitors is far more intricate than previously anticipated, and that imbalances in the pervasive and highly connected interaction network they form part of can have unexpected but far-reaching consequences [107].

While TIMP-1 has long been known to be overexpressed in PDAC [108–112], it has only recently become apparent that TIMP-1 strongly influences tumor progression by triggering pro-metastatic conditioning of the liver. An initial hint at an important role for TIMP-1 in hepatic PMN formation has been the finding that elevated systemic levels of TIMP-1 promote the scattered infiltration of T lymphoma cells throughout the parenchyma of the liver [106, 107, 113]. Strikingly, TIMP-1 overexpression even redirects the spread of human fibrosarcoma cells from the lungs, where they typically form large metastatic foci, to the liver [106], implying that TIMP-1 specifically primes the liver microenvironment and thus dictates the pattern of metastasis in an organ-specific manner. This may be one reason why pancreatic cancer cells exhibit a predilection for metastasis to the liver.

Fundamental insights into how TIMP-1 prepares the liver microenvironment for the influx of tumor cells in PDAC have emerged only recently, largely owing to the elegant work of Grünwald and colleagues [114]. Perhaps the most intriguing aspect of their work is the finding that the process of hepatic PMN development in PDAC is set in motion exceptionally early. TIMP-1 is already produced and secreted into the circulation by pancreatic premalignant lesions, and even patients with chronic pancreatitis, an inflammatory lesion from which PDAC may also develop, exhibit upregulated expression of TIMP-1. These observations suggest that a pro-metastatic niche is formed in the liver not only long before dissemination of pancreatic cancer cells, but already before (and in parallel with) primary tumor formation in the pancreas.

TIMP-1-induced conditioning of the liver for metastatic progression of PDAC is independent of its N-terminal protease-inhibitory function. When pancreas-derived TIMP-1 reaches the liver via the circulation, its C-terminal domain serves a signaling role by binding to CD63, a tetraspanin that is abundantly expressed on quiescent hepatic stellate cells (HSCs). The interaction of TIMP-1 with CD63 then initiates HSC activation. Since activated HSCs themselves are a major source of TIMP-1, this simultaneously generates an autoregulatory feedback loop that perpetuates HSC activation. Subsequent production and secretion of SDF-1 by activated HSCs establishes a chemotactic gradient along which neutrophils can infiltrate the liver via CXCR4. A previous study demonstrated that the TIMP-1-induced accumulation of neutrophils in the liver can be ascribed to enhanced granulopoiesis rather than direct mobilization or prolonged survival of mature neutrophils, as reflected by a 3-day delay in neutrophil recruitment and enrichment of myeloid progenitors in the bone marrow [115]. Neutrophils play an eminent role in PMN development as they actively promote ECM remodeling, angiogenesis and the chemoattraction of other inflammatory cells, each of which will subserve future pancreatic cancer cell colonization. Moreover, secretion of SDF-1 by activated HSCs has been demonstrated to attract colorectal carcinoma cells to the liver via CXCR4 [116]. TIMP-1-induced production of SDF-1 in the liver could potentially represent one mechanism directing the organotropic metastasis of pancreatic cancer cells, which also express high levels of CXCR4 [117]. Upregulation of TIMP-1 expression has also been identified as a potential resistance mechanism of pancreatic tumors against gemcitabine, and has been implicated in clonogenic survival of pancreatic cancer cells as well as vascular density [118].

In light of these findings, it is not surprising that high serum levels of TIMP-1 correlate with poor prognosis in patients with PDAC [119]. Plasma levels of TIMP-1 are higher in patients harbouring liver metastases compared to those who do not manifest metastatic disease [114]. Furthermore, Poruk and colleagues have demonstrated the utility of measuring systemic TIMP-1 levels as a diagnostic tool to distinguish early, resectable PDAC from healthy tissue and chronic pancreatitis [119]. Since chronic pancreatitis has been identified as a risk factor for PDAC [120], TIMP-1 warrants further attention as a potential biomarker to stratify patients with chronic pancreatitis based on their risk of pancreatic cancer.

#### Pancreatic cancer cell-shed exosomes: Cell-free messengers of metastasis

Beyond conventional signaling driven by soluble factors such as TIMP-1 and direct cell-cell contact, intercellular communication may also be mediated by more complex mechanisms involving small membrane-bound vesicles known as exosomes. Exosomes are a type of extracellular vesicle ranging from 30 to 150 nm in size that are actively shed by a multitude of cells, including malignant cells, and serve as vehicles for the horizontal transfer of a diverse array of proteins, lipids and nucleic acids [121]. Though exosomes are key mediators of intercellular signaling in normal physiology, it is rapidly becoming clear that they play a pivotal role in various cancer-related processes, particularly in the evolution of the PMN [122]. The secretion of exosomes enriched in functional biomolecules not only allows pancreatic cancer cells to shape the activity of adjacent cells in the tumor microenvironment, but also allows them to exploit distantly located cells in order to optimize the conditions for future metastatic seeding.

Costa-Silva et al. have recently defined several sequential exosome-mediated events that drive the development of the hepatic PMN in PDAC [121]. The relevance of these vesicular messengers in metastasis was corroborated by the finding that exosomes derived from the murine PDAC cell line PAN02 caused an increase in metastatic burden in the liver when injected into wildtype mice. Immunofluorescence and flow cytometric analysis showed that the vast majority of PDAC-derived exosomes selectively fuse with Kupffer cells, specialized macrophages that reside in the liver. Exosomes have been shown to be able to govern organotropic metastasis owing to specific repertoires of integrins expressed on their surface [123]. Exosomes shed by pancreatic cancer cells express the integrin α_v_β_5_ and thus interact specifically with Kupffer cells. Following adhesion, integrin uptake by Kupffer cells elicits upregulation of pro-migratory and pro-inflammatory *S100P* and *S100A8* genes, suggesting that the interaction of exosomal integrins with recipient cells can trigger niche formation. In this way, PDAC-derived exosomes are able to direct the metastatic dissemination of pancreatic cancer cells to the liver.

One of the most highly upregulated genes following the fusion of PDAC-derived exosomes with Kupffer cells is the gene encoding TGFβ, a well-established mediator of liver fibrosis [121]. TGFβ secreted by Kupffer cells acts directly on hepatic stellate cells (HSCs) to stimulate their activation, leading to enhanced deposition of the ECM component fibronectin. The fibronectin-rich ECM induced by PDAC-derived exosomes provides a docking site for metastatic tumor cells, as well as for macrophages and myeloid-derived suppressor cells mobilized from the bone marrow. Their arrest in pre-metastatic sites may be mediated by the presence of fibronectin-binding α_4_β_1_ (VLA-4) and α_4_β_7_ (LPAM-1) integrins expressed on their cell surface, as is the case for bone marrow-derived hematopoietic progenitors expressing vascular endothelial growth factor receptor 1 (VEGFR1) [19]. As discussed in a later section, the homing of bone marrow-derived cells to future metastatic sites is a centrally important aspect of PMN formation [19, 124]. This is emphasized by the finding that depletion of fibronectin abolishes the pro-metastatic phenotype of PAN02 tumors in vivo [121]. Kupffer cells with upregulated expression of TGFβ can be detected remarkably early on during the development of PDAC, indicating that PDAC-derived exosomes play a pivotal role in the initial stages of PMN formation.

Costa-Silva et al. were able to identify macrophage migration inhibitory factor (MIF) as one of the molecular constituents of pancreatic cancer cell-derived exosomes that are integral to the construction of a permissive niche in the liver [121]. MIF was found to be expressed at high levels in PDAC-derived exosomes, and genetic ablation of MIF prevented the sequential events involved in liver PMN formation and pancreatic cancer metastasis in mice. This pro-inflammatory cytokine has previously been reported to contribute to pancreatic cancer aggressiveness by inducing EMT and reducing chemosensitivity in pancreatic cancer cell lines [125]. Given that increased expression of MIF in pancreatic exosomes is an early event which precedes liver metastasis in PDAC patients, MIF could potentially serve as a prognostic factor for metastatic risk [121]. The prognostic value of MIF in PDAC is further strengthened by the fact that elevated intratumoral levels of MIF mRNA correlate with poor survival in PDAC patients who have undergone surgery [125].

The increased awareness of the pervasive influence of exosomes on PDAC progression has sparked an enormous initiative to investigate their use as potential markers for the early detection of PDAC. Additionally, carbohydrate antigen 19–9 (CA 19–9), the only biomarker for PDAC that has been approved by the US Food and Drug Administration (FDA) to date, has proven to be a poor diagnostic tool [126]. The presence of elevated levels of CA 19–9 in non-malignant lesions such as peptic ulcers, chronic and acute pancreatitis and cirrhosis limits its utility as a biomarker [127–129]. Besides, CA 19–9 has inadequate sensitivity to detect early-stage PDAC, as a mere 50% of patients bearing pancreatic tumors less than 3 cm in size exhibit increased levels of CA 19–9 in the serum [129]. Table 1 displays several exosomal nucleic acids and proteins which have emerged as promising screening tools that may complement or replace CA 19–9 and other current diagnostic modalities in the early identification of PDAC. As already demonstrated in the study by Costa-Silva et al. [121], exosomal contents may also be of prognostic relevance. Pancreatic cancer patients with distant metastatic disease were found to have higher levels of circulating glypican-1 (GPC1)^+^ exosomes than those with regional or no metastasis, and a substantial decrease in GPC1^+^ exosomes in the serum of PDAC patients following resection could predict improved overall and disease-specific survival [130]. Lastly, integrin expression profiles on circulating tumor exosomes may also be clinically relevant in predicting sites of future metastasis [123].

**Table 1:** Candidate exosomal biomarkers for the early detection of PDAC. miR, microRNA; CD44v6, cluster of differentiation variant 6; Tspan8, tetraspanin-8; EpCAM, epithelial cell adhesion molecule; CD104z, cluster of differentiation 104z; KRAS, Kirsten rat sarcoma virus; TP53, tumor protein p53

Exosomal marker	Diagnostic value	Sensitivity	Specificity	Reference
Glypican-1 (GPC1)	GPC1 is a specific marker of cancer cell-derived exosomes and exosomal GPC1 levels can distinguish patients with early- and late-stage PDAC from healthy subjects and patients with benign pancreatic lesions	100%	100%	[130]
High miR-10b, miR-21, miR-30c, and miR-181a and low miR-let7a	Differentiates pancreatic cancer cases from healthy and chronic pancreatitis cases	100%	100%	[191]
CD44v6, Tspan8, EpCAM, MET, CD104z, miR-1246, miR-4644, miR-3976, miR-4306	Combined evaluation distinguishes patients with PDAC from healthy subjects and patients with non-malignant disease	100%	80%	[192]
miR-17-5-p	Discriminate between pancreatic cancer patients and healthy subjects	72.7%	92.6%	[193]
miR-21	Discriminate between pancreatic cancer patients and healthy subjects	95.5%	81.5%	[193]
*KRAS*, *TP53*	Exosomes contain genomic DNA with mutated *KRAS* and *TP53*	–	–	[194]

### Systemic immune cell recruitment

Beyond local changes such as an increase in vascular permeability, extensive remodeling of the ECM and manipulation of liver-resident cell types, the hepatic PMN in PDAC is also shaped by systemic changes pertaining to the immune system. Like the initial acquisition of invasive properties, intravasation and extravasation, continuous immune surveillance forms yet another rate-limiting step in the metastatic cascade that needs to be overcome to ensure successful metastatic outgrowth of incipient pancreatic cancer cells. As such, pancreatic cancer cells have evolved a myriad of mechanisms by which they can evade host immune responses. This section will cover ways in which the primary tumor in PDAC can mold pre-metastatic sites into immunosuppressive environments.

#### Metastasis-associated macrophages create a supportive niche while warding off tumor-targeting immune cells by inducing fibrosis

It is widely recognized that macrophages are actively recruited to the microenvironment of the primary tumor where they foster disease progression by promoting angiogenesis, migration and intravasation, and repressing anti-tumor immune responses [131, 132]. However, in recent years there has also been a growing appreciation for macrophages that populate distant (pre-)metastatic lesions and influence metastatic outcome at these sites. In a recent report, Nielsen et al. emphasized the significance of a distinct population of macrophages termed metastasis-associated macrophages (MAMs), which assist hepatic metastasis in PDAC by establishing a fibrotic microenvironment in the liver [133].

MAMs originate from inflammatory monocytes which undergo differentiation following recruitment from the bone marrow to the liver. The chemotactic factors that initiate their recruitment were not evaluated in the study by Nielsen et al., but potential candidates include C-C motif chemokine ligang 2 (CCL2) and SDF-1 secreted by pancreatic cancer cells [134, 135]. Once infiltrated in the liver, soluble factors derived from pancreatic cancer cells, for example colony stimulating factor-1 (CSF-1) [136], trigger MAMs to produce and secrete granulin, a glycoprotein previously implicated in the wound healing response and breast cancer as a potent activator of stromal fibroblasts [137, 138]. Consistent with this role, macrophage-derived granulin instigates the activation of HSCs into myofibroblasts during liver metastasis in PDAC [133]. Unfortunately, our current mechanistic understanding of granulin-mediated HSC activation is limited due to conflicting reports with respect to the cognate cell-surface receptor for granulin. Nonetheless, granulin-activated HSCs are known to secrete high levels of ECM components, giving rise to a highly fibrotic environment. Besides collagen, one of the proteins particularly enriched in activated HSCs is periostin. This matricellular protein has previously been reported to encourage metastatic tumor development in colon cancer by augmenting cell survival via the Akt/PKB pathway [139]. In another study, breast cancer stem cells were shown to induce periostin expression in fibroblasts to aid metastatic colonization, presumably because deposition of periostin creates a supportive microenvironment which resembles that of the primary niche. Furthermore, periostin was found to support stem cell maintenance by facilitating Wnt signaling, thereby allowing breast cancer stem cells to initiate metastatic growth [140]. Nielsen et al. extended this work by demonstrating the requirement of periostin in the survival and growth of pancreatic cancer cells in vitro; a neutralizing antibody against periostin abolished the stimulatory effects of myofibroblast-conditioned medium on colony formation and proliferation of pancreatic cancer cells [133]. Notably, induction of periostin expression in activated HSCs was found to be strictly regulated by MAM-derived granulin, and depletion of granulin in KPC mice significantly diminished metastatic growth of pancreatic cancer cells. These findings further illustrate the central role of granulin and, consequently, periostin in PDAC metastasis.

Though this newly-described mechanism supporting liver metastasis in PDAC may seem reminiscent of the role of Kupffer cells, Nielsen et al. propose that these liver-resident macrophages and MAMs exert distinct functions within the temporal sequence of events [133]. While Kupffer cells are believed to predominantly facilitate initial seeding of pancreatic cancer cells, MAMs appear to be of greater relevance to subsequent cancer cell survival and growth. This may lead one to argue that MAMs are not involved in the early stages of PMN formation in the liver. In fact, it remains unclear from the study by Nielsen et al. whether MAM-induced activation of HSCs and concomitant fibrogenesis actually precede the arrival of pancreatic cancer cells in the liver. One methodological limitation of this study is the use of an experimental model of metastasis, which bypasses primary tumor development and hence fails to recapitulate the cardinal features of PMN formation [141]. In this regard, spontaneous models of metastasis are considered the gold standard. Further studies employing these models are therefore anticipated in order to gain a more comprehensive and spatiotemporal understanding of how MAMs assist colonization of pancreatic cancer cells in the liver.

Either way, the pro-metastatic role of granulin-secreting MAMs in vitro and in vivo is consistent with clinical observations in PDAC patients correlating inflammatory monocyte density in the peripheral blood with decreased patient survival [134]. Moreover, since granulin expression is already elevated in circulating inflammatory monocytes from KPC mice as well as from PDAC patients harboring liver metastases, it could have potential utility as a biomarker to predict metastasis in PDAC [133]. In this respect, it would be worth assessing whether granulin is also upregulated in inflammatory monocytes from patients with premalignant or inflammatory lesions. If this is the case, it would greatly increase the potential of granulin as a predictive biomarker.

In a recent extension of this work, Schmid’s group uncovered an additional pro-tumorigenic function of MAMs that may causally link the abundance of inflammatory monocytes and decreased patient survival [135]. Macrophages in general display a high degree of plasticity [132]. In response to environmental stimuli, they can be polarized into a variety of phenotypes, with immune-stimulatory M1 and immunosuppressive M2 macrophages at either end of the spectrum. Initial seeding of pancreatic cancer cells and the development of micrometastases is accompanied by infiltration with tumoricidal cytotoxic T cells and M1-like MAMs, but upon progression to overt macrometastatic lesions, M2-like MAMs predominate, resulting in loss of T cell infiltration and effector function [135]. Since the efficacy of immune checkpoint blockade is heavily dependent on the ability of cytotoxic T cells to infiltrate into tumors, this rendered metastatic lesions less responsive to treatment with an anti-PD-1 monoclonal antibody. Functional experiments in PDAC mice revealed that CSF-1, which is abundantly expressed by metastatic pancreatic cancer cells, is able to expand the population of MAMs while driving their differentiation toward an M2-like phenotype.

CSF-1 also induces granulin expression by MAMs. Conceivably, the resulting desmoplastic reaction forms a physical barrier that precludes T cell infiltration into the metastatic site, thereby protecting pancreatic cancer cells from tumor-targeting immune responses. In line with this theory, residual cytotoxic T cells were noted to cluster primarily in peripheral regions of anti-PD-1-resistant tumors, in close proximity to activated HSCs [135]. Furthermore, the reduction in the number of cytotoxic T cells correlated with an accumulation of activated HSCs as well as enhanced collagen deposition. Interruption of metastasis-associated hepatic fibrosis by means of CSF-1 blockade or granulin depletion restored T cell infiltration and sensitized metastatic tumors to anti-PD-1 therapy in vivo. These data suggest that, via the release of CSF-1, metastatic pancreatic cancer cells are capable of undermining cytotoxic T cell-mediated immune surveillance by exploiting M2-polarized MAMs to construct a dense fibrotic stroma in the liver. Most importantly, they provide a strong rationale to explore the therapeutic benefit of immune checkpoint inhibitors in combination with compounds targeting granulin, and perhaps other pro-fibrotic factors, in advanced PDAC.

#### Myeloid-derived suppressor cells: Imposing immune tolerance

Our understanding of tumor immunology and the importance thereof has expanded rapidly over the years and has spawned a renewed surge of interest in another population of bone marrow-derived cells termed myeloid-derived suppressor cells (MDSCs) [142]. MDSCs are a heterogeneous population of incompletely matured cells of myeloid origin endowed with potent immunosuppressive activity. In conditions of chronic inflammation and cancer, myelopoiesis is persistently stimulated which impairs the normal differentiation of granulocyte/monocyte precursors into mature granulocytes, monocytes or dendritic cells, leading to the accumulation of phenotypically similar but functionally distinct MDSCs in the peripheral blood, lymphoid organs and tumor-bearing tissues [142, 143]. Indeed, numerous studies have reported increased prevalence of circulating MDSCs in patients with various types of cancer, for example breast cancer [144, 145], non-small cell lung cancer [144], head and neck carcinoma [144], colorectal carcinoma [146, 147], renal cell carcinoma [148] and PDAC [145, 149, 150].

It is likely that MDSCs originally evolved as safeguards against uncontrolled immune responses that may damage healthy tissues [143]. Tumors, however, cunningly take advantage of this protective function and actively recruit MDSCs to permit tumor development and progression in the absence of tumor-specific immune attacks. Though a multitude of mechanisms have been described through which MDSCs perturb anti-tumor immunity, which immunosuppressive mechanism predominates seems to be partly influenced by the specific nature of the MDSC subset that is expanded. MDSCs can be broadly divided into granulocytic MDSCs and monocytic MDSCs. The prevalence of either of these two subsets differs depending on cancer type, though the currently available literature suggests that patients with PDAC accumulate both granulocytic and monocytic MDSCs in the peripheral blood and pancreatic tumor tissue [142, 151–153].

Granulocytic MDSCs release high levels of arginase I, an enzyme that reduces the availability of l-arginine in the microenvironment and in the circulation by hydrolyzing l-arginine to l-ornithine and urea [151, 154]. l-arginine is required for T cell proliferation, cytokine production and expression of the ζ chain of the T cell co-receptor molecule CD3 (CD3ζ), so deficiency of this amino acid causes profound T cell dysfunction [148]. Simultaneously, arginase I directly subserves tumor growth since the newly generated supply of l-ornithine can be used for the synthesis of polyamines, compounds critically implicated in cell growth and survival [155, 156]. The reactive oxygen species (ROS) hydrogen peroxide (H_2_O_2_) derived from granulocytic MDSCs systemically suppresses T cell function in a similar manner [157]. In addition, ROS released by MDSCs mediate antigen-specific cytotoxic T cell tolerance by disrupting the integrity of the T cell receptor (TCR) complex [158]. MDSCs have the ability to take up foreign antigens, process them and present the resulting peptides to T cells. By mediating the nitration of tyrosine residues within the TCR complex and causing its dissociation, MDSC-derived ROS interfere with the interaction between MDSCs and cytotoxic T cells and prevent the induction of a T cell response against the presented peptide [158, 159]. In a genetically engineered mouse model that spontaneously develops PDAC, tumor infiltration with MDSCs was found to be almost mutually exclusive with the loss of intratumoral effector T cells, indicative of a strong inhibition of T cell function and proliferation [160]. In agreement with this observation, targeted depletion of granulocytic MDSCs in KPC mice was shown to reestablish accumulation of activated cytotoxic T cells within the pancreatic tumor, accompanied by apoptosis of tumor cells and remodeling of the tumor stroma [161].

While T cells are their prime targets [143], MDSCs have also been reported to negatively regulate NK cell function by inducing NK cell anergy via membrane-bound TGF-β1 [162, 163]. Other studies have implicated MDSCs in the recruitment and maintenance of regulatory T cells (T_reg_), a subpopulation of T cells renowned for their role in sustaining tolerance to self-antigens and in restricting excessive immune responses [160, 164]. Since many tumor antigens are in fact self-antigens, T_reg_ are also involved in limiting anti-tumor immune responses. Therefore it is not surprising that T_reg_ have been noted with increased prevalence in multiple forms of cancer, including PDAC [165]. Moreover, MDSCs have been shown to support neovascularization, providing further evidence that they also directly favor tumor progression. To this end, MDSCs may either secrete pro-angiogenic factors such as bombina variegate peptide 8 (Bv8) [166], VEGF or basic fibroblast growth factor (bFGF) [167], or liberate VEGF from the tumor-associated stroma by means of MMP9 [168, 169]. MDSCs may even actively participate in tumor angiogenesis by differentiating into endothelial-like cells capable of embedding in the growing vascular endothelium [168].

While the association between MDSCs and the establishment of a PMN in the liver has not been thoroughly explored in PDAC, there is ample evidence to speculate that MDSCs indeed prepare the hepatic microenvironment for metastatic spread of pancreatic cancer cells by means of their pro-tumorigenic, immunosuppressive activities. Accumulation of MDSCs in the peripheral blood positively correlates with metastatic tumor burden in patients with PDAC and other types of cancer [145], and ablation of MDSCs by means of a neutralizing antibody profoundly suppresses metastasis [170]. Studies using a variety of tumor models, including a mouse model of PDAC, have revealed the capacity of MDSCs to home to and expand in the liver during pre-metastatic phases [171, 172]. Importantly, depletion of liver MDSCs in mice harboring extrahepatic primary tumors was shown to dramatically reduce the frequency of liver metastases, indicating that these hepatic populations of MDSCs accelerate metastatic tumor growth in the liver [172]. Here, MDSCs were found to inhibit cytotoxic T cell activation, proliferation and cytotoxicity, as well as induce the development of T_reg_. Furthermore, MDSCs within the liver have been demonstrated to interact with Kupffer cells and augment their expression of the negative T cell costimulatory molecule PD-L1 [171]. Notably, amplification of MDSCs can be detected in pancreatic tissue and in the peripheral blood of mice even before fully established primary tumors manifest in the pancreas [173]. Whether MDSCs also have a direct effect on circulating pancreatic cancer cells, however, has not been investigated.

The above findings suggest that early tumor-mediated recruitment of MDSCs to the future metastatic site in the liver ensures prosperous metastatic outgrowth by shielding incoming pancreatic cancer cells from tumor-specific immunosurveillance. Accordingly, numerous reports have identified distinct mechanisms through which pancreatic cancer cells elicit the accumulation of MDSCs. Keratinocyte-derived chemokine (KC), the murine homolog of human C-X-C motif ligand 1 (CXCL1), is a soluble factor that is already secreted by precursor lesions in a murine model of PDAC [172]. Ligation of KC to the KC receptor (CXCR2 in humans) is required for the previously described expansion of MDSCs in the livers of tumor-bearing mice. Mice deficient in the KC receptor showed a reduction in hepatic MDSC accumulation by more than 75%, while primary tumor growth remained unaffected. In the liver, MDSCs themselves secrete high levels of KC, among other regulatory and pro-inflammatory cytokines, so as to maintain the hepatic MDSC population. Consistent with these findings, Steele et al. [170] noted greatly upregulated expression of CXCL1 in KPC mice. Inhibition or genetic deletion of its receptor CXCR2 nearly completely abrogated metastasis in this model. Depletion of MDSCs, which are naturally a prominent source of CXCR2, had a similarly profound effect on metastasis. These data indicate that KC/CXCL1-dependent recruitment of MDSCs to the liver is indeed a pivotal event in the formation of a hospitable PMN.

Other soluble factors that have been associated with the recruitment of MDSCs by pancreatic cancer cells include granulocyte/macrophage colony-stimulating factor (GM-CSF) [174] and pancreatic adenocarcinoma upregulated factor (PAUF), a soluble protein previously implicated in pancreatic cancer metastasis [175]. PAUF has been suggested to attract MDSCs to tumor sites in an SDF-1-dependent manner by upregulating the expression of CXCR4 on MDSCs. In addition, the tumor-associated stroma may also interact with MDSCs to encourage their expansion and enhance their function; a recent study demonstrated the capability of PSCs to secrete several cytokines and chemokines that promoted the differentiation of peripheral blood mononuclear cells into immunosuppressive MDSCs, including IL-6, VEGF and macrophage colony-stimulating factor (M-CSF), and SDF-1 and monocyte chemoattractant protein-1 (MCP-1), respectively [176].

Interestingly, pancreatic cancer cell-derived exosomes have also been implicated in skewing the immune repertoire toward an immunosuppressive kind. Exosomes shed by pancreatic cancer cells can be internalized by CD14^+^ monocytes and impart a monocytic MDSC phenotype by downregulating HLA-DR expression and activating signal transducer and activator of transcription 3 (STAT3) signaling, with increased arginase I and ROS production as a result [153]. Another study reported that exosomes secreted by pancreatic cancer cells could expand both granulocytic and monocytic populations of MDSCs while lowering the number of dendritic cells, and proposed altered intracellular calcium fluxes as a potential mechanistic explanation [177]. The specific contents of pancreatic cancer cell-derived exosomes that govern the observed reprogramming of monocytes is presently unclear. Still, because exosomes have previously been shown to be employed by the primary tumor in PDAC to distantly prepare the liver for metastasis, these exciting discoveries provide further evidence supporting a role for MDSCs in the formation of a hepatic PMN in PDAC.

## Conclusions

As highlighted in this review, the dynamic interplay between pancreatic cancer cells and their microenvironment strongly contributes to metastatic success. PSCs and matrix stiffness augment the invasive capacity of pancreatic cancer cells through a variety of mechanisms, in which the induction of EMT appears to be a recurrent theme. Two exciting emerging concepts warranting attention are the potential ability of PSCs to co-migrate with pancreatic cancer cells to distant organs to facilitate their seeding, and the ability of pancreatic cancer cells to co-opt stiffened collagen fibers as migratory tracks. However, metastasis is a highly complex process and goes far beyond mere dissociation of malignant cells from the primary tumor. Both in vitro and in vivo studies have clearly demonstrated that crosstalk among the primary tumor, platelets, HSCs, Kupffer cells and bone marrow-derived cells rigorously modulates the liver microenvironment such that it favors colonization by infiltrating pancreatic cancer cells. The early induction of PMN formation may begin to explain the capacity of pre-neoplastic pancreatic epithelial cells to enter the circulation and colonize distant organs [83] and, with that, the exceptionally aggressive nature of PDAC. This challenges the axiom that metastasis is a linear process involving the gradual evolution of a benign, local lesion into a malignant tumor [178], and lends support to a parallel progression model [179].

Accumulating evidence supports a link between TF-induced platelet aggregation and PMN formation. Strikingly, even though high intratumoral TF expression has been identified as an adverse prognostic factor in PDAC [180, 181], clinical studies so far have failed to find a correlation between TF expression and hematogenous metastasis [181]. This is disconcerting because it has previously been demonstrated that inhibition of TF completely abolished hematogenous metastatic spread in a murine model of PDAC [182]. In another study, increased microparticle-associated TF activity in the plasma of pancreatic cancer patients was only present in those with metastatic, non-resectable tumors [94]. Further analyses are required to reconcile these discrepancies regarding the clinicopathological significance of TF.

A largely unexplored question in this field is what determines the organ specificity of TIMP-1 in PMN development. While TIMP-1 is known to promote liver metastasis, it actually reduces metastasis to the lungs [106]. Similarly, various studies have documented organ-specific promotion of metastasis upon inhibition or overexpression of proteases or protease families [183–185]. Additional studies dissecting the complexities of the pro-metastatic effects of TIMP-1 are therefore highly anticipated. Still, the recent discoveries implicating TIMP-1 in the evolution of the hepatic PMN could potentially open new avenues from a therapeutic standpoint. If rising plasma levels of TIMP-1 indeed represent a reliable biomarker of metastatic progression in PDAC, they might provide a window of opportunity for intervention with, or even prevention of, liver metastasis. However attractive this approach might seem theoretically, the therapeutic targeting of TIMP-1 would also entail important mechanistic issues. As described earlier, excessive proteolytic activity can remodel the ECM in favor of invasion. Straightforward inhibition of TIMP-1 might in fact advance the spread of pancreatic cancer cells as a consequence of reduced protease inhibition. The identification or development of a compound that abrogates the ability of TIMP-1 to interact with CD63 while preserving its protease-inhibitory function may overcome this issue. Clearly, further work is required in this area before clinical applications can be considered.

Exosomes are emerging as key players in intercellular communication and niche formation in PDAC. MIF has proven to be essential in this process, but requires further validation as a prognostic biomarker, which calls for clinical studies that link elevated levels of MIF in patients with chronic pancreatitis to the development of liver metastases. Undoubtedly, exosomes impact pancreatic cancer metastasis in ways that reach beyond MIF-dependent interactions. Proteomic and gene ontology enrichment analyses of exosomal cargo revealed that many proteins enriched in pancreatic cancer exosomes are functionally involved in metabolic processes [186], suggesting that exosome-mediated metabolic reprogramming may represent an additional aspect of PMN formation. Given the present paucity of reliable diagnostic biomarkers of PDAC, efforts have been made to harness the potential of exosomes to aid the early detection of pancreatic tumors. Exosomes are abundant and stable, and their isolation is cheaper and less invasive than current diagnostic methods. Sadly, the present heterogeneity in techniques used to isolate exosomes curtails the clinical relevance of laboratory-based studies of exosomes [187]. Isolation methods will need to be standardized and streamlined to allow high-throughput purification and analysis of exosomes in the clinic. Large-scale prospective studies are also required to validate candidate exosomal biomarkers in a population-screening context and to confirm that early detection of PDAC indeed improves the likelihood of cure, but such studies are thwarted by the limited availability of biospecimens from patients with early-stage disease caused by the current lack of early detection markers [188]. Evidently, the road to successful implementation of exosomes as diagnostic biomarkers will likely be a long one.

Suppression of the immune system also proved integral to the evolution of the PMN in the liver, yet many gaps remain in our mechanistic understanding of the contribution of MAMs and MDSCs to PMN formation. The functional role of MAM-derived periostin in PDAC metastasis and the tumor-secreted factors that regulate MDSC activity remain speculative. Still, the preliminary insights discussed here are hoped to serve as building blocks for further research into the interaction between pancreatic cancer cells and immunosuppressive cells.

Lastly, the studies reviewed here are limited by the use of different preclinical models to examine PMN formation, some of which fail to exemplify the complex cellular and molecular architecture of the PMN [189, 190]. The development of in vivo models that recapitulate PMN formation more faithfully is an important future direction in the area of PDAC metastasis, as they will allow researchers to further dissect the tumor-host interactions that regulate the evolution of the hepatic niche in PDAC, as well as the spatial and temporal organization of these interactions. Notwithstanding the above shortcomings, the novel insights discussed in this review hold promise to help surmount the current lack of therapeutic success in PDAC.

## Abbreviations

bFGF: basic fibroblast growth factor
Bv8: Bombina variegate peptide 8
CA 19–9: Carbohydrate antigen 19–9
CAF: Cancer-associated fibroblast
CCL2: C-C motif chemokine ligand 2
COX-2: Cyclooxygenase 2
CSF-1: Colony stimulating factor-1
CXCL1: C-X-C motif ligand 1
CXCR4: C-X-C chemokine receptor 4
ECM: Extracellular matrix
EMT: Epithelial-to-mesenchymal transition
FAK: Focal adhesion kinase
FDA: Food and Drug Administration
FGF2: Fibroblast growth factor 2
FGFR1: Fibroblast growth factor receptor 1
FVII: Coagulation factor VII
GM-CSF: Granulocyte/macrophage colony-stimulating factor
GPC1: Glypican 1
HAS2: Hyaluronan synthase 2
hPSC: Human pancreatic stellate cell
HSC: Hepatic stellate cell
IL: Interleukin
KC: Keratinocyte-derived chemokine
MAM: Metastasis-associated macrophage
MCP-1: Monocyte chemoattractant protein-1
M-CSF: Macrophage colony-stimulating factor
MDSC: Myeloid-derived suppressor cell
MIF: Macrophage migration inhibitory factor
MMP: Matrix metalloproteinase
NK: Natural killer
PAUF: Pancreatic adenocarcinoma upregulated factor
PD-1: Programmed cell death protein 1
PDAC: Pancreatic ductal adenocarcinoma
PDGF: Platelet-derived growth factor
PD-L1: Programmed death ligand 1
PMN: Pre-metastatic niche
PSC: Pancreatic stellate cell
PTGS2: Prostaglandin-endoperoxide synthase 2
ROS: Reactive oxygen species
SDF-1: Stromal-derived factor-1
STAT3: Signal transducer and activator of transcription 3
TCR: T cell receptor
TF: Tissue factor
TGFβ1: Transforming growth factor β1
TIMP-1: Tissue inhibitor of metalloproteinases-1
T_reg_: Regulatory T cell
VEGF: Vascular endothelial growth factor
VEGFR1: Vascular endothelial growth factor receptor 1
αSMA: α-smooth muscle actin
